# Novel Chromogenic Medium CHROMagar^TM^ Candida Plus for Detection of *Candida auris* and Other *Candida* Species from Surveillance and Environmental Samples: A Multicenter Study

**DOI:** 10.3390/jof8030281

**Published:** 2022-03-09

**Authors:** Juan Vicente Mulet Bayona, Carme Salvador García, Nuria Tormo Palop, Amparo Valentín Martín, Carmelo González Padrón, Javier Colomina Rodríguez, Javier Pemán, Concepción Gimeno Cardona

**Affiliations:** 1 Department of Microbiology and Parasitology, Consorcio Hospital General Universitario de Valencia, 46014 Valencia, Spain; carme.salvador1@um.es (C.S.G.); nuriatormo@hotmail.com (N.T.P.); concepcion.gimeno@uv.es (C.G.C.); 2 Department of Microbiology and Parasitology, Hospital Universitari I Politècnic La Fe de Valencia, 46026 Valencia, Spain; valentin_amp@gva.es (A.V.M.); peman_jav@gva.es (J.P.); 3 Department of Microbiology and Parasitology, Hospital Clínico Universitario de Valencia, 46010 Valencia, Spain; melo-tny@hotmail.com (C.G.P.); jcolominarodri@yahoo.es (J.C.R.); 4 Department of Microbiology and Ecology, University of Valencia, 46010 Valencia, Spain

**Keywords:** *Candida auris*, yeasts, multi-drug resistant, surveillance

## Abstract

Epidemiological trends show a dramatic increase in the prevalence of fungal infections, and in the isolation of multidrug-resistant species, such as *Candida auris.* CHROMagar^TM^ Candida (CC; CHROMagar, Paris, France) and other chromogenic media, which are widely used in the clinical laboratory because they allow a rapid identification of most *Candida* species. Recently, CHROMagar^TM^ Candida Plus (CC-Plus; CHROMagar, Paris, France) was developed to detect and differentiate *C. auris* in addition to other major clinical *Candida* species, such as *C. albicans*, *C*. *tropicalis*, *C. glabrata*, or *C. krusei. C. auris colonies* display a differential light blue color with a blue halo. A multicentric study was designed to evaluate the performance of the CC-Plus medium in the detection of *Candida* species in patients’ surveillance and environmental samples from three Spanish hospitals with active *C. auris* outbreaks. A total of 364 patients’ surveillance samples and 212 environmental samples were tested. Samples were inoculated in CC and CC-Plus in parallel, and the plates were read at 24 and 48 h. All recovered colonies were presumptively identified according to colony color described by manufacturer, and the definitive identification was performed by mass spectrometry at 48 h. A total of 134 *C. auris* isolates were obtained (101 from patients’ surveillance samples, and 33 from environmental samples). Sensitivity, specificity, and predictive positive and negative values were 99.5%, 100%, 100%, and 99.1%, respectively, for the main clinical *Candida* species, showing that CC-Plus is comparable to CC, with the advantage of being able to differentiate *C. auris* from *C. parapsilosis*. Furthermore, CC-Plus was able to detect one *C. albicans*, one *C. glabrata*, and eight *C. auris* that did not grow in CC. Additionally, the yeast colonies were generally larger, suggesting that this novel medium could be a richer medium, and suitable for surveillance and environmental cultures of *C. auris* and other clinically relevant *Candida* species.

## 1. Introduction

*Candida* species can cause several types of infection, such as oral, esophageal, vulvovaginal, intra-abdominal, and even life-threatening invasive infections, such as candidemia [1,2]. Most of the patients with candidemia are critically ill, and have underlying diseases [3], and mortality rates for these patients are reported to be high: between 40% and 70% [3,4,5,6]. Additionally, epidemiological trends show a dramatic increase in the prevalence of fungal infections, with the identification of new at-risk groups, which is moreover worsened with the fact that the isolation of multidrug-resistant species is rising, as is the case of the emergence of the novel multidrug-resistant pathogenic yeast *Candida auris* [7]. Moreover, some fungal species, such as *C. auris*, have been shown to display good patient-to-patient transmission and prolonged environmental persistence in clinical settings, causing major outbreaks, especially in intensive care units (ICU) [8,9,10,11,12].

The diagnosis of invasive candidiasis (IC) may be delayed due to the absence of pathognomonic symptoms of the disease, and the time required for yeasts to grow [13]. The importance in patient survival of an early correct treatment of candidemia has been widely demonstrated [14,15], and the rise of fluconazole-resistant non-*albicans Candida* species makes the establishment of adequate empiric antifungal regimens difficult [3]. Therefore, it is useful to study the epidemiology and incidence of *Candida* spp. colonization and infections in high-risk units, e.g., intensive care units (ICU) or hematology/oncology units, where the patients have more risk to develop IC, particularly if there is an established outbreak of multidrug-resistant species [16]. In the case of *C. auris*, it is highly recommended to detect colonized patients as a measure of effective infection control practices [9]. In this context, environmental sampling may be also of interest, since several studies have proven that *C. auris* can persist on surfaces for days, suggesting that contaminated surfaces may be a relevant source of acquisition [9,17,18].

Among the different methods developed to detect yeasts in the clinical laboratory [19], chromogenic media are widely used because they allow a rapid presumptive identification compared to non-chromogenic media, and are useful in the detection of mixed cultures [20]. They are helpful for the rapid diagnosis of IC, but also for the epidemiological surveillance in high-risk units. CHROMagar^TM^ Candida (CHROMagar, Paris, France) was the first available commercial medium for the identification of yeasts [21], although it cannot differentiate the emerging yeast *C. auris*, since it grows with a non-specific white to mauve color, requiring further confirmation with complementary methods, e.g., Matrix-Assisted Laser Desorption/Ionization-Time of Flight (MALDI-TOF). CHROMagar^TM^ Candida Plus medium (CHROMagar, Paris, France) has been developed to discriminate *C. auris* colonies among those of other species, since it grows with a specific light blue color with a blue halo [22,23]. Here, we present a multicentric study designed to evaluate the performance of the CHROMagar^TM^ Candida Plus medium in the detection of *Candida* species in surveillance and environmental samples from three Spanish hospitals with active *C. auris* outbreaks.

## 2. Materials and Methods

Samples were collected in three centers with active *C. auris* outbreaks in the city of Valencia, Spain: Consorcio Hospital General Universitario de Valencia (CHGUV), Hospital Universitari i Politècnic La Fe (HUiP La Fe), and Hospital Clínico Universitario de Valencia (HCUV). Two types of samples were collected: patients’ surveillance samples (axillary–rectal, axillary, rectal, pharyngeal, inguinal and nasal swabs, bronchoaspirates (BAS), urine, and tracheal aspirates) and environmental samples. Samples were collected from April to June 2021 by the nursing staff of each hospital room. Patients’ surveillance samples were selected from previously cultured samples to include about 30% of *C. auris*, 60% of non-*auris Candida* spp., and 10% of negative samples to obtain a representative number of each clinically important *Candida* spp. to calculate the sensitivity, specificity, and predictive positive and negative values (PPV and PNV, respectively) of the CC-Plus medium. Patients’ surveillance samples were 100 from CHGUV, 152 from HCUV, and 112 from HUiP La Fe; and environmental samples were 27 from CHGUV, 81 from HCUV, and 104 from HUiP La Fe. The methodology used for patients’ surveillance samples varied slightly depending on the hospital, according to each center protocol. At CHGUV and HUiP, samples were collected at admission of patients in the ICU, then once a week until ICU discharge, whereas in other hospital wards, surveillance was performed to contacts of colonized/infected patients with *C. auris*. In both centers, axillary–rectal, axillary, pharyngeal, nasal, rectal, and inguinal swabs were introduced in liquid Amies transport medium, and then 50 μL were inoculated in CHROMagar^TM^ Candida (CC) and CHROMagar^TM^ Candida Plus (CC-Plus) in parallel. BAS and tracheal aspirates were first homogenized with a vortex, and then 50 μL were inoculated in parallel on both agar plates. In HCUV, surveillance samples were collected at admission, and twice a week in the ICU, and once a week in the hematology/oncology unit. Axillary–rectal, pharyngeal, and nasal swabs were recollected in Amies transport medium, and, unlike the previous two centers, samples were previously incubated at 37 °C for 24 h in Brain Heart Infusion broth (BHI), and then 50 μL were inoculated on CC and CC-Plus agar plates in parallel. Sample types obtained in each center are summarized in Table 1. Environmental samples were collected in the ICU from surfaces (bed/bed rail, bedside table, computer keyboard/mouse, and perfusion pump) and medical devices (thermometers, stethoscopes, and blood pressure cuffs) in the setting of colonized/infected patients with *C. auris*. They were collected with wet gauzes or sponges (3M^®^ Sponge Sticks, St. Paul MN, USA) that were introduced in BHI tubes, and then incubated at 37 °C for 48 h, and, after that time, 50 μL were inoculated in CC and CC-Plus in parallel.

All plates were incubated at 37 °C, and they were read at 24 and 48 h. All recovered colonies were presumptively identified according to colony color described by the manufacturer (Table 2), and the definitive identification was performed by mass spectrometry (MALDI-TOF; Bruker, United States) at 48 h. The plates were read independently by two qualified laboratory staff. Size and quantity of the colonies were evaluated. Colony count was evaluated according to the following scale: + (1–10 colonies), ++ (11–30 colonies), +++ (31–50 colonies), ++++ (>50 colonies). Sensitivity, specificity, PPV, and PNV were calculated for CC-Plus compared to CC as a reference medium. A quality control of each batch of plates was performed with the following ATCC^®^ control strains: *Candida albicans* ATCC^®^ 60193, *Candida auris* ATCC^®^ MYA-5001, *Candida tropicalis* ATCC^®^ 1369, *Candida krusei* ATCC^®^ 14243, *Candida glabrata* ATCC^®^ 2001, *Escherichia coli* ATCC^®^ 25922.

## 3. Results

A total of 576 samples (364 patients’ surveillance samples, and 212 environmental samples) were tested. Reading of the plates at 24 h showed that it was not enough time to correctly determine the species according to the color of the colony without using complementary methods, as most of them were colorless. Only in some cases, a bluish color could be perceived for *C. auris* on CC-Plus if the culture was pure and abundant. The isolated species identified by mass spectrometry at 48 h are shown in Table 3. In 41 samples (19.3%), a mixed culture was obtained in either of the two media. All colonies were generally larger on CC-Plus (with an average size of 2.5 mm on CC-Plus, and 2.2 mm on CC), and the size was significantly larger with few colonies on the plate. Colony count was similar in both plates except for 40 samples: more colonies were obtained in CC-Plus in 24 samples (4.2%), whereas in 16 samples, the count was higher in CC (2.8%). For *C. auris*, the colony count was greater in CC-Plus in eight samples, whereas in three samples, it was the opposite: higher in CC.

*C. auris* was isolated in the following surveillance samples: axillary–rectal (46/153; 30.1%), axillary (22/28; 78.6%), inguinal (15/24; 62.5%), rectal (6/24; 25.0%), pharyngeal swabs (5/89; 5.6%), BAS (3/8; 37.5%), urine (3/5; 60.0%), and nasal swab (1/21; 4.8%). As for the environmental samples, a total of 33 *C. auris* were isolated (15.6%) in bed/bed rail (12 positive samples), medical devices (8), bedside table (7), computer keyboards/mice (3), and perfusion pumps (3). In four environmental samples, *C. auris* was detected in CC-Plus, with a colony count of 1–10 yeast colonies, but showed no growth in CC. *C. albicans* was isolated from bed/bed rail (three positive samples), bedside table (two), medical devices (one), perfusion pump (one), and keyboard (one). *C. glabrata* was isolated from bed/bed rail (three positive samples), perfusion pump (two), bedside table (one), and medical devices (one). *C. parapsilosis* was isolated from a bedside table. A total of 167 environmental samples (78.8%) yielded a negative result for any *Candida* spp. *C. albicans* was the dominant species (144/576; 25.0%), followed by *C. auris* (134/576; 23.3%), *C. glabrata* (84/576; 14.6%), *C. tropicalis* (19/576; 3.3%), *C. parapsilosis* (15/576; 2.6%), and *C. krusei* (8/576; 1.4%) as the most clinically important species. Sensitivity, specificity, PPV, and PNV of CC-Plus for these species, taking CC as a reference medium, are shown in Table 4. Furthermore, CC-Plus was able to detect one *C. albicans*, one *C. glabrata*, and eight *C. auris* more than CC. These cultures had a colony count of 1–10 yeast colonies in CC-Plus, and showed no growth in CC. These samples corresponded to four patients’ surveillance samples, and four environmental samples. Five samples were from CHGUV, and three were from HCUV. Five of these samples were mixed cultures.

**Figure 1:**
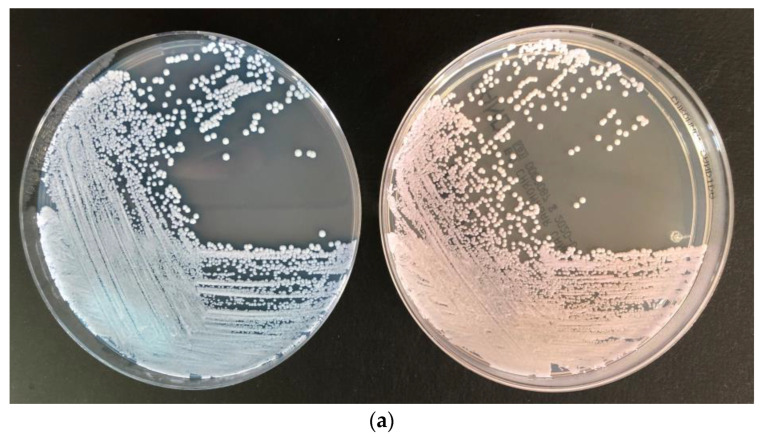

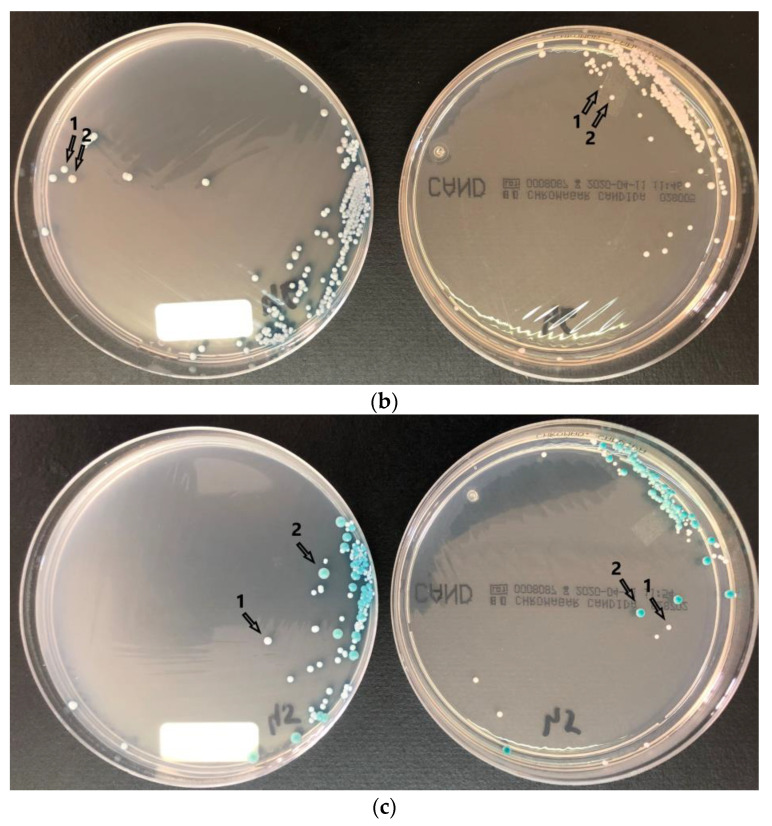
Patients’ surveillance samples inoculated directly on CC-Plus (left) and CC (right) plates. The plates were incubated for 48 h. The recovered colonies were first presumptively identified according to the colony color, and the definitive identification of all different colonies were performed with MALDI-TOF. Colors were as described by the manufacturer. A larger colony size can be observed in CC-Plus plates, especially for *C. auris*. (**a**) Axillary–rectal sample from CHGUV with *C. auris* in CC-Plus (left) and CC (right) (**b**) Axillary–rectal sample from CHGUV with *C. auris* (1) and *C. parapsilosis* (2) in CC-Plus (left) and CC (right). Although these species have a similar bluish color in CC-Plus, a blue halo can be observed in the case of *C. auris*. In CC, by contrast, *C. auris* and *C. parapsilosis* cannot be differentiated. (**c**) Pharyngeal sample from CHGUV with *C. auris* (1) and *C. albicans* (2) in CC-Plus (left) and CC (right).

Colony colors in CC-Plus at 48 h were as described by the manufacturer, except in the following cases: in three samples, the blue color of *C. auris* was described as less intense; in two samples, *C. auris* could not be properly distinguished because it was mixed with a large amount of *C. albicans*; in one sample with *C. auris* and *C. parapsilosis*, the colors could also not be accurately distinguished; and in two samples, *C. parapsilosis* had a purplish–blue color which may be confused with *C. tropicalis*. However, these colonies could be correctly identified by MALDI-TOF. Examples of the plates are shown in Figure 1.

## 4. Discussion

A rise in the prevalence of fungal infections has been observed, which might be a result of the expanding number of patients at risk, notably those with an impairment of their immune response, such as HIV-infected people, transplant recipients, patients on immunomodulators, premature neonates, and the elderly [24]. Moreover, recently, the COVID19 pandemic has also been linked to an increase in fungal infections [25,26,27]. Furthermore, the increasing emergence in multidrug-resistant species, such as *C. auris*, makes advisable to study the incidence and epidemiology of candidemia in high-risk units, where the patients have more risk to develop invasive infections, and the nosocomial pathogens can spread easily.

Effective infection control practices and screening for colonization are crucial to contain an outbreak caused by *C. auris* [9]. In fact, occult colonization has been shown to facilitate the persistence of outbreaks involving other pathogens, such as *Klebsiella pneumoniae* [28]. There are still limited published data about the relative sensitivity of different body sites to detect *C. auris*, although it has been reported that at least six body sites would be necessary to detect all colonized patients [29]. However, sampling six body parts is not affordable for large-scale routine screening, and it is recommended to target high-yield sites [29]. Axillae and groins are typically screened, as Public Health England (PHE) pointed them as the most persistently positive body parts [30]. More recently, a higher sensitivity has been reported for anterior nares [31]. Including additional sampling sites may be of interest in cases of persistent outbreaks, as well as performing environmental sampling. In our study, it can be observed that there exists much heterogeneity in the body sites that are screened for colonization in the different centers; although they all include axillae and groins, as recommended by PHE, or the rectum as an adjacent body part to groins. Although *C. auris* positive samples were previously selected, it can be seen that axillary, rectal, and inguinal swabs were the most positive samples, as expected. In respect to environmental samples, there is little information available about the extent of contamination of surfaces in healthcare facilities, and their role in the transmission of *Candida* species, although it is known that *Candida* species can survive for prolonged periods on surfaces [32]. Some studies found that *C. auris* does not have a greater propensity to survive on surfaces than other *Candida* species, whereas other investigations conclude that *C. auris* remains viable for at least 14 days [17,33]. In our study, *C. auris* was found in 33 environmental samples (15.6%): in beds/bedrails, medical devices (including thermometers, stethoscopes, and blood pressure cuff), bedside tables, computer keyboards and mice, and perfusion pumps, reinforcing the hypothesis that the environment might be an important source of transmission.

Early diagnosis is key for the successful treatment of fungal infections, and more rapid and reliable tests are needed [34,35]. Non-culture-based methods, such as PCR, are rapid methods to diagnose yeast infections, although their use in clinical laboratories is still low, especially in resource-limited settings [36]. Chromogenic media are a valuable alternative in resource-limited settings due to their lower costs and ease of use, allowing a rapid presumptive identification of the main clinical *Candida* species [20]. CHROMagar^TM^ Candida Plus facilitates the isolation and presumptive identification of major clinically important yeast species through a chromogenic technology. For instance, *C. albicans* produces beta-N-acetylgalactosaminidase, a green chromogenic hexosaminidase substrate is incorporated directly into the growth medium, and *C. albicans* isolates are spotted directly on primary isolation as green colonies. The main difference between CC and the new version, CC-Plus, is the differentiation of *C. auris* with a specific morphology, different from the morphology of any other *Candida* species. CC-Plus performed well in comparison to CC, with the advantage to be able to differentiate *C. auris* from *C. parapsilosis*. Furthermore, CC-Plus was able to detect one *C. albicans*, one *C. glabrata*, and eight *C. auris* that did not grow in CC. The number of *C. auris* that did not grow in CC is significant, considering that the inoculation was the same for both media. This observation could be explained with CC-Plus being a medium designed to detect *C. auris*. All the cultures that did not detect *C. auris* in CC had a colony count of 1–10 colonies in CC-Plus, and five were mixed cultures, showing more sensitivity in the detection of *C. auris* in samples with low fungal loads and/or mixed cultures, which makes CC-Plus suitable for use in detecting colonized patients and contaminated surfaces. Additionally, 24 (4.2%) samples yielded more colonies of *C. auris* in CC-Plus, whereas in 16 samples, the count was higher on CC (2.8%), reinforcing that CC-Plus might be a richer medium for *C. auris*. It is also noteworthy that the use of slightly different methodologies of sample processing and culture between the three hospitals did not affect the performance of CC-Plus. The methodology in HCUV included an initial incubation of the samples in BHI, which might presumably increase the growth of yeasts, although no major differences were observed between the three centers, and more studies should be performed to establish the best procedure in sample processing for *C. auris* surveillance studies. Although, in some samples, the colors in CC-Plus were not exactly as described by the manufacturer; this occurred especially in mixed cultures, and the colonies could be detected and correctly identified by MALDI-TOF. The same limitations were found for these samples on CC.

Apart from clinical samples, rapid and reliable methods are also necessary in surveillance studies to establish infection control practices, to anticipate an IC, and to help in directing antifungal treatment, due to the emergence of multidrug-resistant yeasts, such as *C. auris*. Whereas in clinical samples it is recommended to confirm the identification with complementary methods, an advantage of using CC-Plus in surveillance samples is that complementary methods could be avoided (always by keeping periodic control of performance of the plates, and confirming colonies with ambiguous colors), taking into account that a screening method should be simple, inexpensive, and suitable for a large number of determinations. The fact of not requiring a confirmatory method to identify *C. auris* also makes this medium suitable for resource-limited settings where MALDI-TOF is not available.

The limitations of this study are that the protocols followed by the three centers were not unified, that patients’ surveillance samples were previously selected to include about 40% of *C. auris* positive cultures, and that environmental samples yielded many negative cultures. Also, some species genetically related to *C. auris* were not isolated, such as *C. haemulonii* and *C. duobushaemulonii*; although previous studies reported that they were easily distinguishable from *C. auris* in CC-Plus [22]. The advantages of this study are the large sample size, the multicentric methodology, and that real prospective samples from patients and also environmental samples in centers with active *C. auris* outbreaks were used.

## 5. Conclusions

CC-Plus is a useful chromogenic medium for the detection of the most common *Candida* species, with the main advantage over CC of being able to differentiate *C. parapsilosis* and *C. auris*. Moreover, the fact of not requiring a confirmatory method to identify *C. auris* also makes this medium suitable for resource-limited settings where MALDI-TOF is not available. Furthermore, CC-Plus detected eight *C. auris* isolates that did not grow on CC, and the yeast colonies were generally larger, suggesting that this novel medium might be a richer medium, and suitable for samples with low fungal loads, as screening samples of patients, and surfaces suspected of being contaminated with *C. auris.* CC-Plus is, therefore, a valuable method for controlling the outbreaks caused by the nosocomial multidrug-resistant yeast *C. auris*.

## Figures and Tables

**Table 1:** Patients’ surveillance sample types by center.

	Axillary–Rectal ^a^	Axillary ^a^	Rectal ^a^	Pharyngeal ^a^	Inguinal ^a^	Nasal ^a^	BAS	Urine	Tracheal Aspirate
CHGUV	x			x					
HUiP La Fe		x	x	x	x	x	x	x	x
HCUV	x			x		x			x

^a^ Swabs were collected in Amies transport medium.

**Table 2:** Colony color of the main clinical *Candida* species in CHROMagar^TM^ Candida Plus and CHROMagar^TM^ Candida, according to manufacturers.

Species	Color at 36–48 h, 30–37 °C, CHROMagar^TM^ Candida Plus	Color at 48 h, 35–37 °C, CHROMagar^TM^ Candida
*C. albicans*	Turquoise blue/green	Green
*C. krusei*	Pink to purple with white edges	Pink, fuzzy
*C. glabrata*	Pink to purple	Mauve–brown
*C. tropicalis*	Metallic blue with pink halo	Metallic blue
*C. auris*	Light blue with blue halo	White to mauve
*C. parapsilosis* complex	Light blue	White to mauve
*C. lusitaniae*	Pink to purple	White to mauve

**Table 3:** Isolated species identified by mass spectrometry at 48 h.

	Surveillance Samples (*n* = 364)		Environmental Samples (*n* = 212)		Total	
	CC-Plus	CC	CC-Plus	CC	CC-Plus	CC
*C. albicans*	135	135	8	8	143 ^a^	143 ^a^
*C. glabrata*	76	77	7	6	83 ^b^	83 ^b^
*C. tropicalis*	19	19	0	0	19	19
*C. parapsilosis*	14	14	1	1	15	15
*C. auris*	101	97	33	29	134	126
*C. kefyr*	1	1	0	0	1	1
*C. inospicua*	2	2	0	0	2	2
*C. krusei*	8	8	0	0	8	8
*C. dubliniensis*	2	2	0	0	2	2
*Kodamaea ohmeri*	2	2	0	0	2	2
Bacteria	2	2	0	0	2	2
Total number of isolates	362	359	49	44	411	403
Negative	51	55	167	170	218	225

^a^ In one of the patients’ surveillance samples, *C. albicans* grew on CC, but not in CC-Plus, whereas in another sample, it was the opposite (*C. albicans* grew on CC-Plus, but not in CC). Therefore, the total number of *C. albicans* in either of the two media is 144. ^b^ In one of the patients’ surveillance samples, *C. glabrata* grew on CC, but not in CC-Plus, whereas in a surveillance sample, it was the opposite (*C. glabrata* grew on CC-Plus, but not in CC). Therefore, the total number of *C. glabrata* in either of the two media is 84.

**Table 4:** Sensitivity, specificity, PPV, and PNV of CC-Plus for the most common isolated *Candida* species, compared with CC.

	Sensitivity (%)	Specificity (%)	PPV (%)	PNV (%)
*C. albicans*	99.3	100	100	99.8
*C. glabrata*	98.8	100	100	99.8
*C. tropicalis*	100	100	100	100
*C. parapsilosis*	100	100	100	100
*C. krusei*	100	100	100	100
*C. auris*	100	100	100	100
Total	99.5	100	100	99.1

## Data Availability

The data presented in this study are available on request from the corresponding author.
